# Supplementary material for: Learning the heterogeneous hypermutation landscape of immunoglobulins from high-throughput repertoire data
Source: Nucleic Acids Res. 2020 Oct 9;48(19):10702–12. doi: 10.1093/nar/gkaa825 (PMC7641750; doi:10.1093/nar/gkaa825)
Supplement: gkaa825_Supplemental_Files [file gkaa825_supplemental_files.zip › si_nar-.pdf]

Supplementary material for “Learning the heterogeneous hypermutation landscape of immunoglobulins from high-throughput repertoire data”

Natanael Spisak, Aleksandra M. Walczak,* and Thierry Mora*
Laboratoire de physique de l'École normale supérieure, CNRS, PSL University, Sorbonne Université, and Université de Paris, 75005 Paris, France

*Corresponding authors. These authors contributed equally.

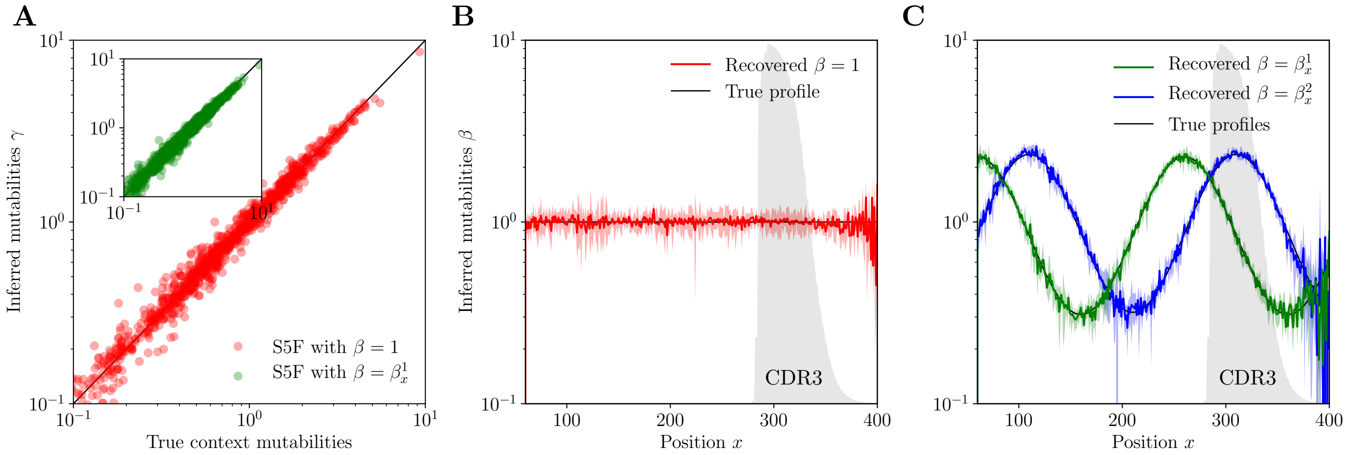

FIG. S1: Model inference on synthetic data using true phylogenies. A. Inference of context mutabilities γ. B,C. Inference of position mutabilities β for flat and sinusoidal profiles. Error bars correspond to 95% confidence intervals. Frequency at which a given position belongs to the CDR3 region is indicated with the grey shaded areas.

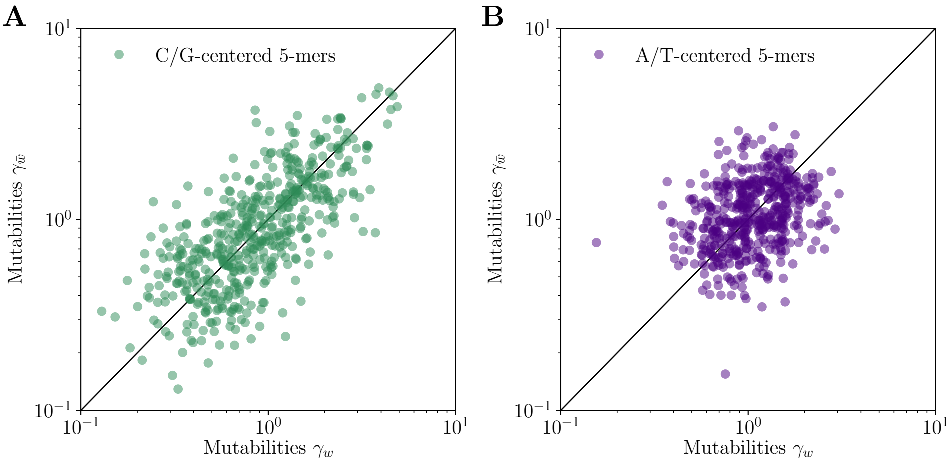

FIG. S2: Strand asymmetry of the context-dependent rates. For each motif w we juxtapose its mutability γ_w with the mutability of its reverse complement $\gamma_{\bar{w}}$. A. 5-mer motifs with strong central nucleotide (C/G), $r^2 = 54\%$. B. 5-mer motifs with weak central nucleotide (A/T), $r^2 = 7\%$

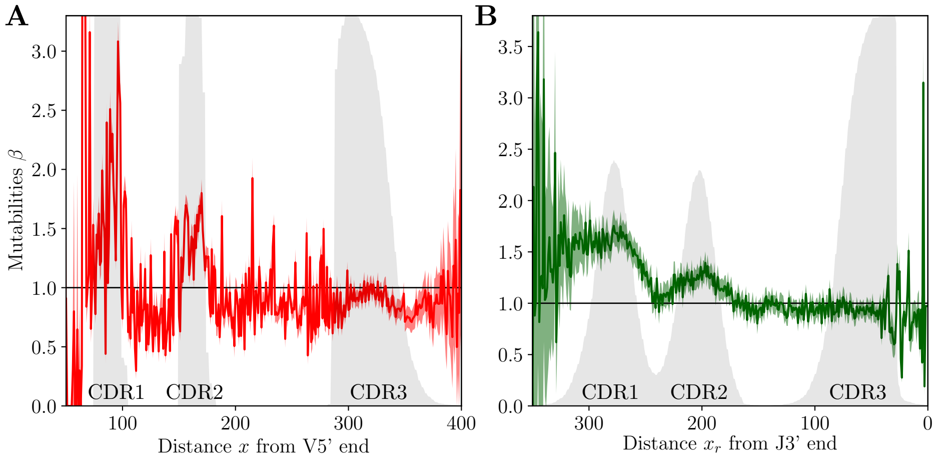

FIG. S3: Alternative position definition. We compare the models based on different position definitions: x, the distance from the $5'$ end of the V gene (A, red) and x_r, distance from the $3'$ end of the J gene (B, green). Error bars correspond to 95% confidence intervals. Frequency at which a given position belongs to a CDR region is indicated with the grey shaded areas.

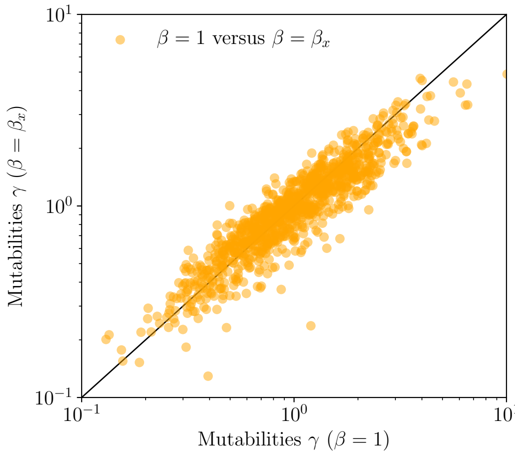

FIG. S4: Introducing explicit position dependence $\beta \neq 1$ influences the context-dependent rates. We compare the γ mutabilities from the full model with the parameters of the purely context-dependent model.

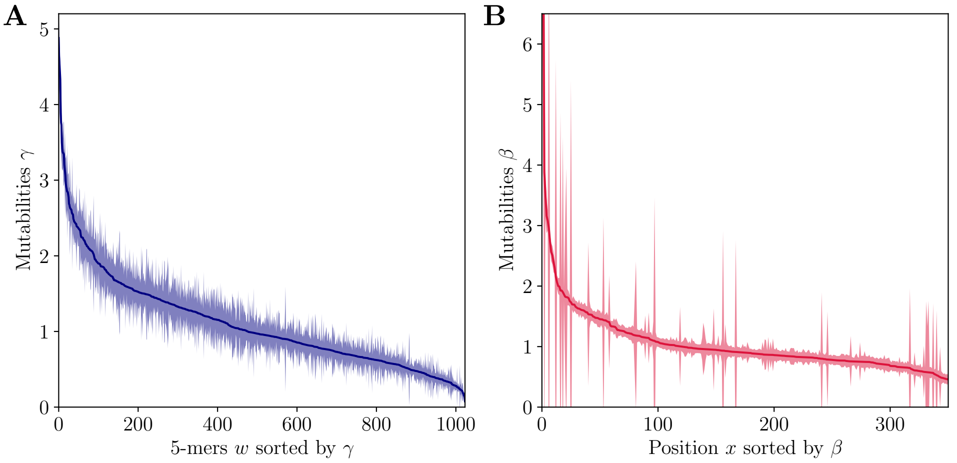

FIG. S5: Analysis of the uncertainty of estimate parameters γ (A) and β (B). The shaded area indicates the 95% confidence interval envelope. Motifs and positions are sorted by their respective mutabilities

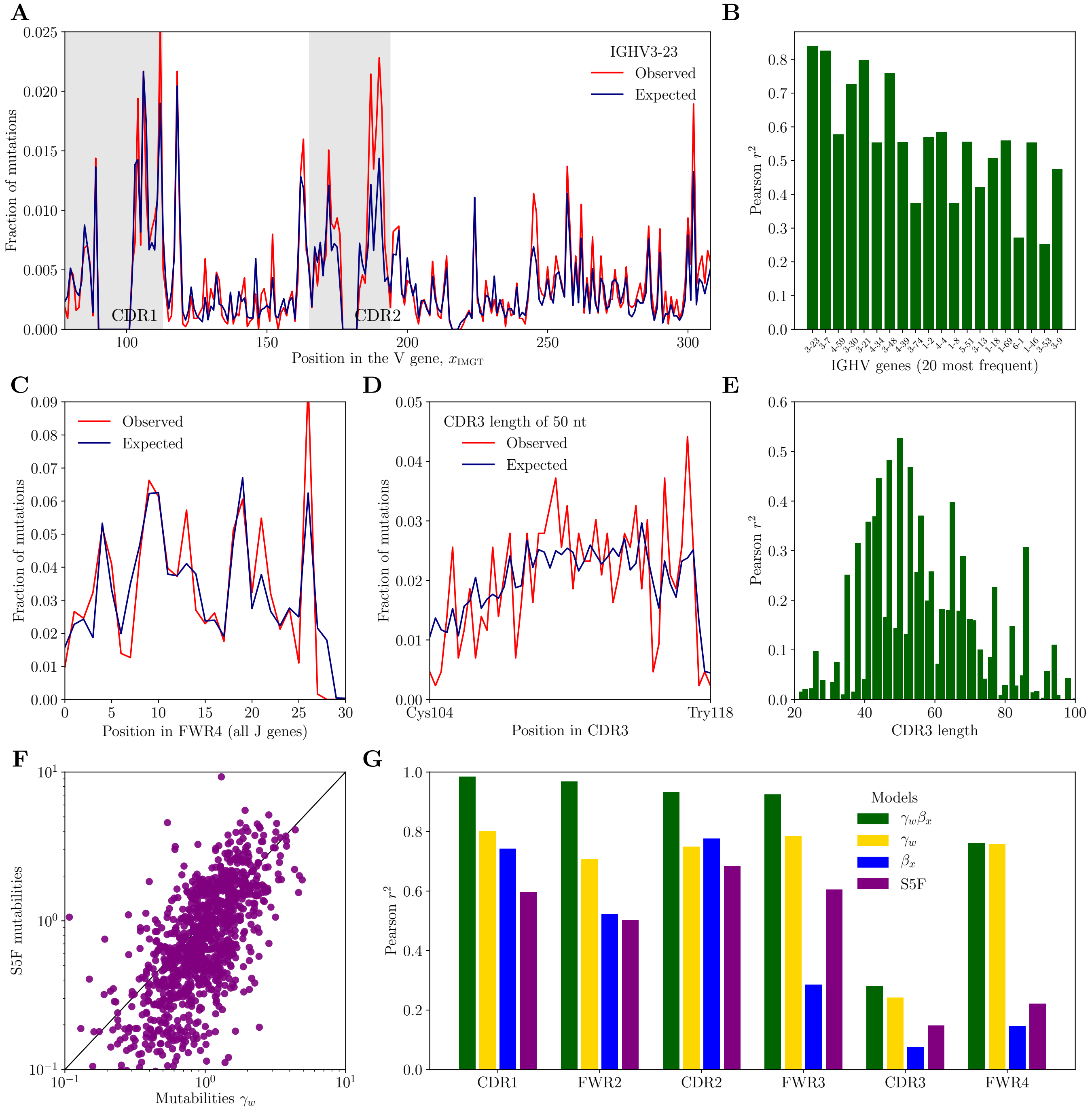

FIG. S6: Model performance when model was trained on 2/3 of data, and tested on the remaining 1/3. A. Example mutation profile in the most common V gene. B. Model performance across V genes. C. Mutation profile in the FWR4 region. D. Example mutation profile in the CDR3 region for CDR3 length of 50 nts. E. Model performance across CDR3 lengths. F. Comparison with the S5F model. G. Summary of models performance across sequence regions.

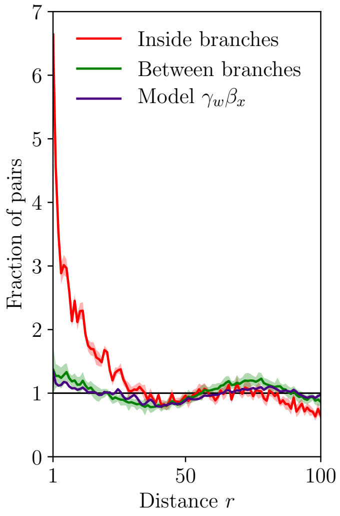

FIG. S7: Fraction of pairs $n(r)$ of mutations encoded in the same tree branches (red) and pairs of mutations between different branches (green) normalized by the total number of pairs at that distance, $(l-r)/\binom{l}{2}$, where l stands for the alignment length. Shaded area corresponds to 95% confidence interval.

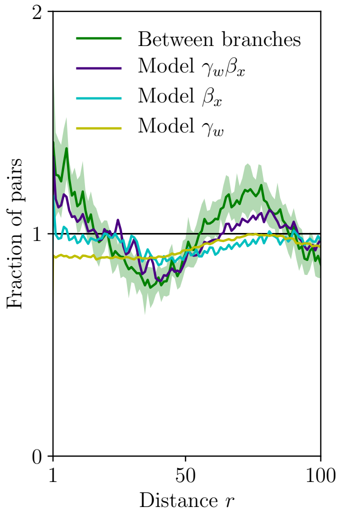

FIG. S8: Normalized fraction $n(r)$ of pairs of mutations between different branches (green). Context- and position-dependent models prediction $n_m(r)$ for this quantity are presented for the full ($\mu_{s,x} = \gamma_w\beta_x$), purely context-dependent ($\mu_{s,x} = \gamma_w$) and purely position-dependent ($\mu_{s,x} = \beta_x$) models. Shaded area corresponds to 95% confidence interval.

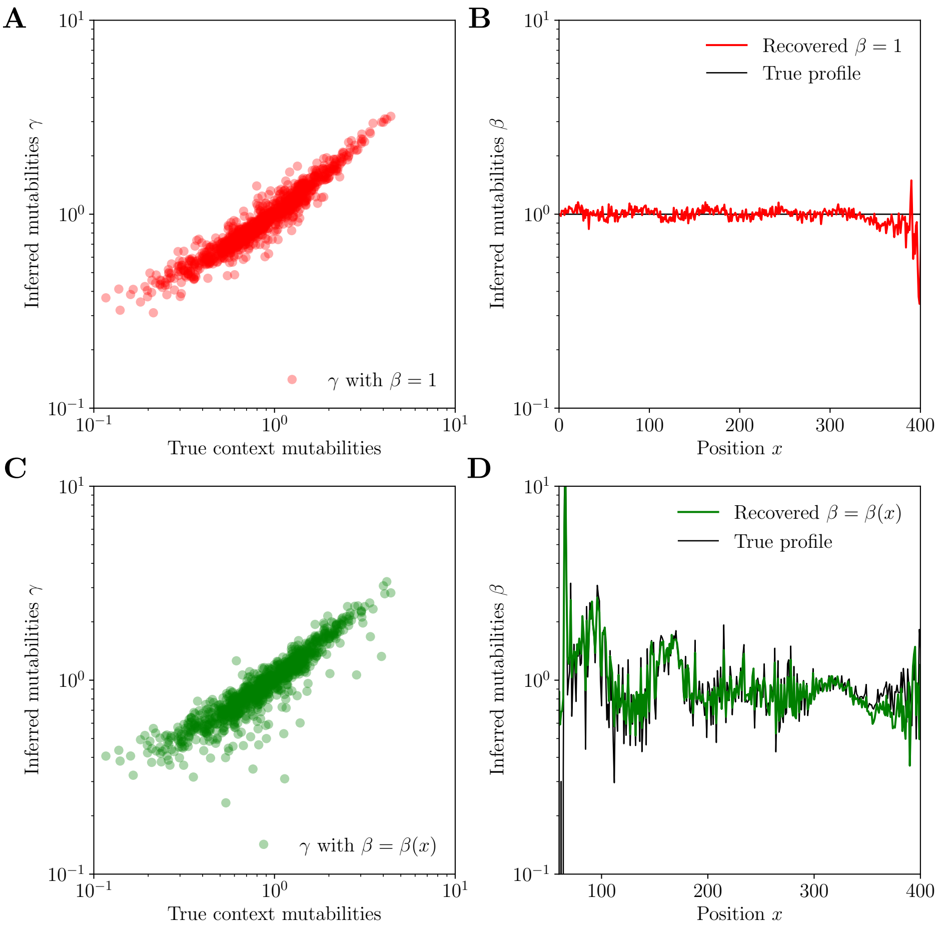

FIG. S9: Model inference on synthetic data with correlated mutations using true phylogenies. Mutations were drawn according to the co-localization model (**??**) with $\epsilon = 5\%$ and $\xi = 10$ with data-derived context and position dependent mutabilities. A,C. Inference of context mutabilities γ. B,D. Inference of position mutabilities β for flat (red, A,B) and data-derived (green, C,D) position-dependent profiles.

TABLE 1: 50 hottest 5-mers w sorted by their mutabilities γ_w.

Rank	5-mer w	Mutability γ_w
1	TAGCT	4.880
2	AGCTC	4.635
3	AAGCT	4.518
4	GAGCT	4.438
5	CAGCT	4.331
6	AGCTA	3.891
7	AGCTT	3.761
8	ACGCT	3.730
9	GGGTT	3.496
10	CAGCA	3.418
11	TAGTT	3.373
12	TAGCA	3.360
13	CGGTT	3.355
14	GAGTA	3.342
15	GTGTA	3.209
16	AGCTG	3.154
17	CGGTA	3.081
18	GTATC	3.056
19	GAGTT	2.955
20	CAAGC	2.910
21	GGGTA	2.887
22	AGCCG	2.852
23	ACGTT	2.849
24	GAGCA	2.839
25	AAGCA	2.811
26	GTAAC	2.775
27	CAGTT	2.686
28	ATATC	2.675
29	TGCTC	2.646
30	AACTT	2.628
31	TACCA	2.623
32	GTATA	2.612
33	TAGTA	2.609
34	GAAGC	2.575
35	TAACG	2.557
36	TGGTA	2.553
37	AAGTA	2.544
38	CAGTA	2.488
39	TCTTC	2.474
40	CCGTA	2.451
41	AACTA	2.448
42	TGCTA	2.437
43	AGCCC	2.431
44	GTAGA	2.425
45	TGCTG	2.422
46	GCGTA	2.393
47	TGGCA	2.386
48	GTACC	2.382
49	GTAGT	2.379
50	GGGCT	2.369

TABLE 2: 50 coldest 5-mers w sorted by their mutabilities γ_w.

Rank	5-mer w	Mutability γ_w
975	TTGGG	0.345
976	ATGGA	0.342
977	GCCGA	0.340
978	CTCAA	0.338
979	ACCGC	0.338
980	GTCCC	0.338
981	AGGGA	0.335
982	TCGGA	0.334
983	GCCCT	0.330
984	GCGGC	0.329
985	CAGAC	0.328
986	GTCAC	0.327
987	GTCAA	0.324
988	AAGGC	0.324
989	TCGCG	0.323
990	GCCCA	0.310
991	TCGAC	0.308
992	ATGGG	0.298
993	CTGGC	0.297
994	CTCCA	0.296
995	CGGTC	0.292
996	CAGGC	0.289
997	GACAC	0.288
998	GCGAG	0.287
999	GCCCC	0.284
1000	GGGGG	0.282
1001	GAGAC	0.281
1002	GACAA	0.275
1003	GGGCC	0.275
1004	CGCGT	0.265
1005	GTCCG	0.262
1006	ACGGC	0.259
1007	GGGGC	0.258
1008	AGGAC	0.257
1009	GCCAG	0.246
1010	AAGGG	0.244
1011	CGCGC	0.237
1012	CGGCC	0.232
1013	GTCCA	0.232
1014	CGGGC	0.227
1015	CTCAG	0.220
1016	TGGGG	0.219
1017	GCGAC	0.212
1018	CTCAT	0.201
1019	GTCGC	0.183
1020	CTGGG	0.177
1021	CCTCA	0.155
1022	GTCGA	0.152
1023	AGGGC	0.129
1024	CGCAA	0.076
